# The Vital Worker: Towards Sustainable Performance at Work

**DOI:** 10.3390/ijerph16060910

**Published:** 2019-03-13

**Authors:** Jan de Jonge, Maria C.W. Peeters

**Affiliations:** 1 Human Performance Management Group, Eindhoven University of Technology, P.O. Box 513, 5600 MB Eindhoven, The Netherlands; m.peeters@uu.nl; 2 Department of Social, Health and Organizational Psychology, Utrecht University, P.O. Box 80140, 3508 TC Utrecht, The Netherlands; 3 School of Psychology, Asia Pacific Centre for Work Health and Safety, University of South Australia, P.O. Box 2471, Adelaide, SA 5001, Australia

**Keywords:** employee vitality, sustainable work performance, thriving at work, work engagement, work redesign, job crafting

## Abstract

Vitality at work is an important factor for organizations to build a healthier, more engaged, sustainable, and productive workforce. The organizational and societal relevance of vitality at work is high, particularly with regard to an aging and more diverse workforce. This Special Issue focusses on what might be called sustainable performance at work: Maximizing work performance as well as worker health and well-being through employee vitality. Currently, there are still many gaps of knowledge with regard to the relationship between employee vitality and sustainable performance at work. Examples of knowledge gaps are concerned with potential determinants of vitality at work for different occupational groups (such as older workers, ethnic minority workers, and handicapped workers), pathways linking vitality to sustainable performance, or health effects of interventions targeting employee vitality and/or sustainable performance at work. With this Special Issue, we hope to provide readers with solid new findings extending the current state of knowledge about employee vitality and sustainable work performance.

## 1. Introduction

Over the last decades, there has been a growing interest in building sustainable organizations. Originally, the concept of sustainability was derived from ecology, referring to the capacity of systems and processes to develop, to grow, to care, and to endure [1,2]. Sustainability is also defined as an effort to conserve, use, and recycle natural resources in an efficient way. The ultimate goal is to ensure that our whole ecosystem will be preserved [3]. How organizations should do their business to protect the environment is called ‘green management’ [4].

So far, most of the interest in the concept of sustainability has been concerned with effects on the physical environment. This raises important questions: What about humans (i.e., employees) and human sustainability? What about managing human resources for sustainability? What are the sustainable consequences of management practices for employee health, well-being, and performance? Finally, how can organizations and management ensure the employee vitality that they need for sustainable work performance? Put differently, which elements of today’s work systems have a strong impact on sustainability? Pfeffer [3] introduced in this respect the overarching term ‘social sustainability’ to reflect how organizational activities and management practices affect employee health, well-being, and performance in a sustainable way.

Sustainability in relation to *work* was first introduced by Docherty et al. in 2002 [5]. They felt a sense-of-urgency that the world of work was moving in a wrong direction. Employees seemed caught in a trap of increasing job demands, decreasing job resources, and decreasing occupational rewards (e.g., [6,7]). They concluded that coming up with an alternative for the future was more important than solely analyzing the past. The authors argued that the future of work sets the stage for two central concepts, that is, intensive and sustainable work systems. *Intensive work systems* can be seen as systems that maintain productivity by depleting human, job, and social resources. They will have damaging effects on both employees and the quality of products and services in the long run [2]. In contrast, *sustainable work systems* are systems where human, job, and social resources are instead regenerated and renewed through the process of work while still maintaining productivity. Sustainable work systems can be considered an important key for sustainable work performance and for maintaining long-term human sustainability. They attempt to stimulate employee vitality, development, and well-being while, at the same time, generating positive and enduring socio-ecological outcomes [8].

### 1.1. Employee Vitality as an Important Aspect of Sustainable Performance

Employee vitality can be considered a crucial aspect of the sustainable work performance concept that is useful for understanding how employee health, well-being, and performance are related to long-term productivity and viability [9]. In the literature, different definitions of vitality are used. According to the Merriam-Webster Dictionary, for instance, vitality refers to a lively and animated character, the power of enduring, the capacity to live and develop, and the peculiarity distinguishing the living from the nonliving. It also refers to physical and/or mental vigor. The Cambridge Dictionary equates vitality to energy and strength. Another definition of vitality is the positive feeling of having energy available to the self [10]. In addition, Ryan and Frederick [11] defined vitality as a specific psychological experience of possessing enthusiasm and spirit. They argued that it is to be related not only to physical health and bodily function, but also to psychological factors such as self-actualization, agency, personal well-being, and growth. Thus, a vital person is energetic and strong, and feels physically and mentally well.

Transferring the concept of vitality to the organizational setting, it is hard to disconnect it from how it is usually measured in empirical studies. Employee vitality is often represented by Shirom’s concept and measurement of vigor [12], as well as by Schaufeli and colleagues’ concept and measurement of work engagement [13]. Shirom considered vigor as employee’s feelings of cognitive liveliness, emotional energy, and physical strength. Thus, he is not only reflecting the mental component of vitality, but also the physical component. To measure it properly, he created the Shirom–Melamed Vigor Measure (SMVM) that was empirically validated in more than 20 empirical studies in different countries [12]. Work engagement is defined as a positive and fulfilling work-related state of mind that is characterized by vigor, dedication, and absorption [14]. Vigor resembles employee vitality, and is characterized by high levels of energy and mental resilience while working, and the willingness to invest effort in one’s work and persistence even in the face of difficulties. To assess work engagement a self-report questionnaire has been developed—the Utrecht Work Engagement Scale (UWES; [13]). Several surveys of global consultancy firms suggest that roughly 25% of the North American workforce can be considered ‘engaged’ [15,16]. In Europe, 21% of European workforce feels ‘engaged’, and about 11% feels ‘highly engaged’ [17]. Managers, executives, farmers, teachers, and artists seem to be most engaged employees, whereas blue-collar workers, police officers, retail workers, and homecare staff seem to be the least engaged [16].

The organizational and societal relevance of vitality at work is high, particularly with regard to the aging and more diverse workforce. Vitality at work is studied in different scientific fields, such as psychology, sociology, organizational behavior, economics, management, and medicine. At present, many researchers in these fields are highly interested in understanding how to optimize employee health and well-being in a sustainable way. For organizations and management, the ultimate aim is to have a staff consisting of vital workers—workers that are happy, healthy, energized, passionate, and engaged. These vital workers are presumed to be highly productive workers as well. In this perspective, sustainable performance at work means maximizing work performance as well as worker health and well-being through vitality at work [18].

Dorenbosch [19,20] created a conceptual framework of sustainable work performance in which employee vitality plays a pivotal role. This framework distinguishes four categories of employees: (1) vital, (2) passive, (3) forced proactive, and (4) comfortable energetic. High vital employees are characterized by high levels of energy, resilience, and proactivity. In sharp contrast, high passive employees have low levels of energy and will not engage in proactive behaviors to improve or adapt their work or work situation. The third category is labeled forced proactive, as this kind of employees experience a strong decline in energy levels which has to be restored by making proactive changes in work or the work situation. Finally, comfortable energetic employees are highly energized but are not investing much effort in proactive behaviors. A cross-sectional survey study among 1966 employees from 13 Dutch organizations empirically confirmed these four categories to a large extent [19].

### 1.2. Thriving at Work as an Important Aspect of Sustainable Performance

Another interesting mechanism for understanding human sustainability and sustainable performance at work is ‘thriving’ [21]. A thriving workforce can be described as one in which employees are not only satisfied and productive, but also actively and continuously seeking out opportunities to learn new things. They are not happy with the current status quo, though highly engaged in shaping the future of their company and their own. Thriving employees are growing, developing, learning, and highly energized, and at the same time they know how to avoid to feel depleted and eventually burned out. Spreitzer and associates [21] identified two components of thriving. The first component is vitality, and denotes the sense of being alive, energized, passionate, and excited at work. Thriving employees have a spark that fuels energy in themselves and other people. Organizations can enhance this by giving them the feeling that what they perform at work makes a huge difference. The second component is learning, which is about growing through new knowledge, skills, and other characteristics. The two components of thriving usually work simultaneously. One cannot exist without the other, and is unlikely to affect sustainable performance. For instance, vitality alone can be boring in the case that work does not result in ample opportunities to learn new things. Learning alone creates momentum for some time, but without energy and passion it can lead to occupational burnout. Porath and her team [22] demonstrated factorial and construct validity of the two-dimensional structure of thriving. Across six industries and different job types, Spreitzer et al. [21] found that employees who fit their description of thriving demonstrated 16% better overall performance (as reported by management) and 125% less self-reported burnout than their peers. In addition, they were 32% more committed to the organization and 46% more satisfied with their jobs. Thriving employees also missed much less work and reported significantly fewer doctor visits, which implied less lost time and money for the organizations. The unique combination of vitality and learning leads to employees who deliver sustainable outcomes and at the same time find ways to grow. Their work is rewarding not just because they successfully perform what is expected of them today, but also because they perform in a sustainable way—having a sense of where they and the organizations are heading to in the longer term. To conclude, thriving as a psychological state in which employees show both vitality and learning behavior contributes to social sustainability [3] and sustainable work performance [18] through physical and psychological well-being.

### 1.3. Determinants of Sustainable Performance at Work 

A final question to be addressed here is how organizations and management can ensure employees’ vitality that they need for sustainable work performance? Or, more specifically, which elements of today’s work systems have the strongest impact on sustainability through vitality and thriving at work? Recent research of the European Foundation for the Improvement of Living and Working Conditions is rather straightforward to this question. According to this research, the most direct and obvious determinant of sustainability at work is *work itself*, operationalized as job quality and usually measured with work characteristics such as job autonomy, skill discretion, career prospects, working time, and a supportive social and physical work environment [2,23]. The danger here is that employees work hard in an intensive work system to maintain productivity while simultaneously depleting their own resources and recovery. In a sustainable work system, key building blocks are the preservation of non-renewable resources and the regeneration of renewable resources [8]. Applied to work itself, it is mainly about sufficient job resources, personal resources, and recovery opportunities to meet job demands [24]. Job resources refer to those physical, psychological, social, or organizational aspects of the job that are either: (1) functional in achieving work goals, (2) reducing job demands and the associated physiological and psychological costs, or (3) stimulating personal growth, learning, and development [24]. Personal resources are aspects of the self that are generally linked to resiliency and refer to employees’ sense of their ability to control and impact upon their work environment successfully [25]. For the regeneration of resources, recovery is considered to be essential [20]. Recovery can generally be defined as a process of unwinding and restoration during which an employee’s functioning and strain level returns to its pre-stressor level [26]. Thus, recovery can be considered as a process opposite to the strain process, in which detrimental effects of stressful situations are at least alleviated or even eliminated. If recovery is successful, employee vitality and performance improve. Thus, the way work is optimally designed in terms of job demands, job resources, personal resources, and recovery supports employees in a sustainable work system. Importantly, employees are not only regarded as recipients of predefined jobs, but also as active and vital people (co-)shaping their own work. In this respect, several authors have posited job crafting as an effective means for employees to shape their own job demands and resources to a large extent [27]. Job crafting can be considered as proactive employee behavior consisting of resource-seeking, challenge-seeking, and demand-reducing that employees engage in to create a better fit with their personal abilities and needs [28]. It also describes how employees cognitively (re-)frame the significance of their work to create more meaningful work [27]. Thus, job crafting might contribute to vitality and thriving at work, and hence sustainable work performance in the long run. Indeed, empirical studies showed evidence for successful job crafting interventions in the workplace (e.g., [29,30]).

## 2. Conclusions

There are still many gaps in the theoretical and empirical knowledge about the relationship between employee vitality and sustainable performance at work. These gaps exist with respect to potential (work) determinants of vitality at work for different occupational groups (such as older workers, ethnic minority workers, and handicapped workers), pathways linking employee vitality to sustainable work performance, or health effects of interventions targeting vitality at work and/or sustainable work performance. With this Special Issue, we hope to provide readers with solid new findings extending the current state of knowledge about employee vitality, thriving, and sustainable work performance. Just as green organizations and green management could enjoy reputational benefits for physical sustainability, we may expect that organizations that take care of human sustainability will enjoy benefits in attracting and retaining vital and thriving employees for lifetime employability and sustainable performance at work.

## References

[1] Holling C.S. (2001). Understanding the complexity of economic, ecological, and social systems. Ecosystems.

[2] Eurofound (2015). Sustainable Work over the Life Course: Concept Paper.

[3] Pfeffer J. (2010). Building sustainable organizations: The human factor. Acad. Manag. Perspect..

[4] Marcus A.A., Fremeth A.R. (2009). Green management matters regardless. Acad. Manag. Perspect..

[5] Docherty P., Forslin J., Shani A.B. (2002). Creating Sustainable Work Systems: Emerging Perspectives and Practice.

[6] Karasek R.A., Theorell T. (1990). Healthy Work. Stress, Productivity, and the Reconstruction of Working Life.

[7] Siegrist J. (1996). Adverse health effects of high-effort/low-reward conditions. J. Occup. Health Psychol..

[8] Kira M., Lifvergren S., Ehnert I., Harry W., Zink K.L. (2014). Sowing seeds for sustainability in work systems. Sustainability and Human Resource Management.

[9] Van Scheppingen A.R., De Vroome E.M.M., Ten Have K.C.J.M., Zwetsloot G.I.J.M., Wiezer N., Van Mechelen W. (2015). Vitality at work and its associations with lifestyle, self-determination, organizational culture, and with employees’ performance and sustainable employability. Work.

[10] Nix G.A., Ryan R.M., Manly J.B., Deci E.L. (1999). Revitalization through self-regulation: The effects of autonomous and controlled motivation on happiness and vitality. J. Exp. Soc. Psychol..

[11] Ryan R.M., Frederick C.M. (1997). On energy, personality, and health: Subjective vitality as a dynamic reflection of well-being. J. Pers..

[12] Shirom A., Perrewe P.L., Ganster D.C. (2004). Feeling vigorous at work? The construct of vigor and the study of positive affect in organizations. Emotional and Physiological Processes and Positive Intervention Strategies Volume 3.

[13] Schaufeli W.B., Bakker A.B., Salanova M. (2006). The measurement of work engagement with a short questionnaire: A cross-national study. Educ. Psychol. Meas..

[14] Schaufeli W.B., Salanova M., González-Romá V., Bakker A.B. (2002). The measurement of engagement and burnout: A two-sample confirmatory factor analytic approach. J. Happiness Stud..

[15] Attridge M. (2009). Measuring and managing employee work engagement: A review of the research and business literature. J. Workplace Behav. Health.

[16] Schaufeli W.B., Salanova M., Peeters M.C.W., De Jonge J., Taris T.W. (2014). Burnout, boredom and engagement in the workplace. An Introduction to Contemporary Work Psychology.

[17] Schaufeli W.B. (2018). Work engagement in Europe: Relations with national economy, governance and culture. Organ. Dyn..

[18] Peeters M.C.W., Taris T.W., De Jonge J., Peeters M.C.W., De Jonge J., Taris T.W. (2014). Introduction: People at work. An Introduction to Contemporary Work Psychology.

[19] Dorenbosch L.W. (2009). Management by Vitality: Examining the ‘Active’ Well-Being and Performance Outcomes of High Performance Work Practices at the Work Unit Level.

[20] Dorenbosch L., Ehnert I., Harry W., Zink K.J. (2014). Striking a balance between work effort and resource regeneration. Sustainability and Human Resource Management.

[21] Spreitzer G., Porath C.L., Gibson C.B. (2012). Toward human sustainability: How to enable more thriving at work. Organ. Dyn..

[22] Porath C., Spreitzer G., Gibson C., Garnett F.G. (2012). Thriving at work: Toward its measurement, construct validation, and theoretical refinement. J. Organ. Behav..

[23] Eurofound (2012). Trends in Job Quality in Europe.

[24] De Jonge J., Demerouti E., Dormann C., Peeters M.C.W., De Jonge J., Taris T.W. (2014). Current theoretical perspectives in work psychology. An Introduction to Contemporary Work Psychology.

[25] Hobfoll S.E., Johnson R.J., Ennis N., Jackson A.P. (2003). Resource loss, resource gain, and emotional outcomes among inner city women. J. Pers. Soc. Psychol..

[26] Sonnentag S., Venz L., Casper A. (2017). Advances in recovery research: What have we learned? What should be done next?. J. Occup. Health Psychol..

[27] Wrzesniewski A., Dutton J.E. (2001). Crafting a job: Revisioning employees as active crafters of their work. Acad. Manag. Rev..

[28] Demerouti E., Bakker A.B., Peeters M.C.W., De Jonge J., Taris T.W. (2014). Job crafting. An Introduction to Contemporary Work Psychology.

[29] Van den Heuvel M., Demerouti E., Peeters M.C.W. (2015). The job crafting intervention: Effects on job resources, self-efficacy, and affective well-being. J. Occup. Organ. Psychol..

[30] Demerouti E., Peeters M.C.W., Van den Heuvel M., Van Zyl L.E., Rothman S. Job crafting interventions: Do they work and why?. Positive Psychological Intervention Design and Protocols for Multi-Cultural Contexts.

